# Integrated behavior and transcriptomic analysis provide valuable insights into the response mechanisms of *Dastarcus helophoroides* Fairmaire to light exposure

**DOI:** 10.3389/fphys.2023.1250836

**Published:** 2023-12-01

**Authors:** Xianglan Jiang, Tengfei Li, Xiaoxia Hai, Xiang Zheng, Zhigang Wang, Fei Lyu

**Affiliations:** ^1^ College of Forestry, Hebei Agricultural University, Baoding, Hebei, China; ^2^ Laboratory of Enzyme Preparation, Hebei Research Institute of Microbiology Co., Ltd., Baoding, Hebei, China

**Keywords:** *Dastartcus helophoroides*, natural enemy, phototactic behavior, nocturnal beetles, transcriptome, ECM-receptor interaction

## Abstract

Light traps have been widely used to monitor and manage pest populations, but natural enemies are also influenced. The *Dastarcus helophoroides* Fairmaire is an important species of natural enemy for longhorn beetles. However, the molecular mechanism of *D. helophoroides* in response to light exposure is still scarce. Here, integrated behavioral, comparative transcriptome and weighted gene co-expression network analyses were applied to investigate gene expression profiles in the head of *D. helophoroides* at different light exposure time. The results showed that the phototactic response rates of adults were 1.67%–22.5% and females and males displayed a negative phototaxis under different light exposure [6.31 × 10^18^ (photos/m^2^/s)]; the trapping rates of female and male were influenced significantly by light exposure time, diel rhythm, and light wavelength in the behavioral data. Furthermore, transcriptome data showed that a total of 1,052 significantly differentially expressed genes (DEGs) were identified under different light exposure times relative to dark adaptation. Bioinformatics analyses revealed that the “ECM-receptor interaction,” “focal adhesion,” “PI3K-Akt signaling,” and “lysosome” pathways were significantly downregulated with increasing light exposure time. Furthermore, nine DEGs were identified as hub genes using WGCNA analysis. The results revealed molecular mechanism in negative phototactic behavior response of *D. helophoroides* under the light exposure with relative high intensity, and provided valuable insights into the underlying molecular response mechanism of nocturnal beetles to light stress.

## 1 Introduction

With global landscape and climate change, insect pests have widespread and frequent outbreaks, seriously damaging agricultural and forest services for human wellbeing, particularly the expansion of invasive pests in nonnative ranges (Scherm et al., 2000; Mina et al., 2022). In many countries, chemical pesticides are still the primary control strategy for pests, but excessive utilization of pesticides has led to problems, such as reducing biodiversity of insects in freshwater and terrestrial systems (Beketov et al., 2013), polluting the environment, increasing pest resistance to pesticides, and contributing to global insect pollinator declines (Basset and Lamarre, 2019). Insect pest management has become one of the biggest challenges, such as on organic farms (Kim et al., 2019). Natural enemies are alternative and environmentally friendly means to control insect pests and have received wide attention worldwide (Landis et al., 2000).

The ectoparasitoid beetle, *Dastartcus helophoroides* Fairmaire (Coleptera: Bothrideridae) is distributed widely in Kyushu and Osaka in Japan, Korea and China (Lyu et al., 2014). This beetle is an important parasitoid of several longhorned beetles in its native range, including *Anoplophora glabripennis* Motschulsky (Haack et al., 2010), *Monochamus alternatus* Hope (Lyu et al., 2014), and *Batocera horsfieldi* Hope (Yang et al., 2014). These longhorn beetles are phytophagous: the larvae usually bore in the large branches and trunks of woody plants, ranging from alive to moribund to dead and decomposing (Hanks, 1999). Many longhorn beetle species cause tremendous economic and ecological losses in forests worldwide, including insect vectors of nematodes (Zhou et al., 2023), such as *A. glabripennis* and the genus *Monochamus*. The *A. glabripennis* is a polyphagous xylophage native to Asia and ranked as one of the top 100 worst invasive species worldwide in North America and Europe (Branco et al., 2022). Potential losses of widespread *A. glabripennis* outbreaks in compensatory value were equal to approximately 12% of the total economic losses caused by forest pests and diseases in China, exceeding more than US$1.5 billion (Golec et al., 2018). Outside its native range, estimations of potential economic losses exceed US$1 trillion if adjusted to 2021 values, and approximately 35% of urban trees have been destroyed in the USA (Branco et al., 2022). Pine wood nematodes is transported between host trees by cerambycid beetles of the genus *Monochamus*, and approximately 1.7 million hectares were infected in 19 provinces in 2021, destroying 14 million trees in China’s pine forests (Yu et al., 2023).

Insects use visual and chemical cues from intra- and interspecific communication and the environment to mediate their activities and behaviors, such as reproduction, and foraging, and even to organize and maintain social structures in social insects (Nieri et al., 2022). Many different stimuli signals have often been applied to pest management, such as chemicals, lights, colors, sounds and vibrations (Fleming et al., 2005; Kim et al., 2019; Cavaletto et al., 2021; Van der Kooi et al., 2021; Zapponi et al., 2022). In cerambycid beetles, pheromone-baited traps are a common tool for detecting and sampling insects for surveillance, eradication, or management, and pheromones of more than 100 cerambycid species have been identified to date, including sex attractant pheromones, aggregation-sex pheromones, and nonvolatile contact pheromones (Hanks and Millar, 2016). However, semiochemical-based traps baited with a mixture of host kairomones (plant volatile organic compounds) and/or sex pheromones have still not reached operational efficacy for some Cerambycid beetles in field bioassays; for example, the trapping number of approximately 80% (34/43) of longhorn beetle species was fewer than 10 beetles per 10–14 days (Nehme et al., 2010; Ray et al., 2012; Wickham et al., 2012; Hanks and Millar, 2013; Wickham et al., 2014; Silva et al., 2016; Wickham et al., 2016; Zhu et al., 2017; Xu et al., 2020) (Supplementary Figure S1). Therefore, an effective trapping tool needs to be developed for some longhorn beetles with limited semiochemical-based trapping effects in the future.

Light traps are widely used to monitor and manage the population density of insect pests, and play an important role in physical pest control (Kim et al., 2019). Due to natural light is present in the daytime, the target pest is usually a crepuscular or nocturnal insect, and at least some of the activity occurs at night (Kim et al., 2019; Nieri et al., 2022). Both foraging and mating behavior in *A. glabripennis* adults occur throughout the day, and the peaking of foraging behavior occurs at night (21:30) (Lyu et al., 2015a). In addition, the mating behavior of *M. alternatus* also took place throughout the day, particularly during the peak time of activity at night (18:00–24:00) (Luo et al., 2012). Longhorn beetles *Phymatodes aereus*, *Anelaphus pumilus*, and *Massicus raddei* are also nocturnal insects (Mitchell et al., 2015). These results suggested that a light-trapping strategy may be used to monitor and manage the population density of Cerambycid beetles. Although light traps can be used to attract and kill insect pest populations, they can also kill the natural enemies of pests (Luo and Chen, 2016). Therefore, the phototactic behavioral responses of natural enemies should be considered when light traps are used to monitor and manage target insect pests.

The phototactic behavior of insects not only depends on the characteristics of the light source, including light wavelength and intensity, but the other conditions also influence phototactic behavior response rates, such as rhythmicity with photoperiod, light exposure time, sex and gene expression (Kim et al., 2019; Kooi et al., 2021). Although the results of previous study showed that near-infrared light (NIR, 700 nm) is most suitable in attracting *D. helophoroides* adults, the phototactic response of *D. helophoroides* have broad spectrum of light (from UV to NIR) and the response rates were not significant difference between UV, blue, green and NIR lights (Wang et al., 2021). In addition, near infrared light (NIR,700 nm) were used to trap Cerambycid beetles has been rarely reported. In the field, UV (or violet), blue, and green lights are usually used to attract and kill target insect pests, including Cerambycid beetles (Luo and Chen, 2016). For example, the beetles (Coleoptera) show a preference for UV, violet (350–440) and green (500–560) wavelength spectra (Kim et al., 2019; Van der Kooi et al., 2021). UV-black-light-blue light traps captured more Cerambycid beetles (*Arhopaus ferus*) than yellow light traps (Pawson et al., 2009). UV (365 nm) and violet light (420 and 435 nm) are the most suitable wavelengths for attracting adult *A. glabripennis* at night (Jiang et al., 2023a). Therefore, in order to decrease the trapping rate of natural enemy (*D. helophoroides*) in the light traps, it is of great significance to reveal the response mechanism of *D. helophoroides* adults for the UV and visible lights.

The behavioral and physiological changes of insects under light exposure are well established (Kim et al., 2019; Kooi et al., 2021) and are tightly related to gene expression and regulation (Futahashi et al., 2015). RNA-Seq is a powerful tool for the analysis of DEGs, the discovery of novel transcripts, and the identification of key genes (Trapnell et al., 2010). It has been widely utilized in various fields ranging from agriculture to medicine, investigating gene expression changes in response to light exposure and other biotic and abiotic stresses (Hong et al., 2020; Chen et al., 2022). In *Diaphorina citri* adults, 841 upregulated differential expressed genes (DEGs) and 932 downregulated DEGs, and DEGs play key roles in physiological and biochemical responses such as oxidative stress, protein denaturation, inflammation and tumor development after blue light (400 nm) exposure (Wang F. F. et al., 2023). While 40 DEGs were upregulated and 413 DEGs were downregulated in the *Bemisia tabaci* after ultraviolet-A radiation, and DEGs were mainly involved in physiological and biochemical responses, including anti-oxidation and detoxification, protein turnover, metabolism (Khan et al., 2023). Many studies have shown that downregulated DEGs are significantly greater than upregulated DEGs after different light exposures and the antioxidant activity is decreased (Cui et al., 2021; Khan et al., 2023). However, these data are insufficient to fully understand the molecular mechanisms underlying the response to light exposure, especially in nocturnal Coleoptera beetles.

In the present study, we investigated the influence of light exposure time, sex, and diel rhythm on the phototactic behavioral response of female and male *D. helophoroides* under light with different wavelengths, and the gene expression profiles of females and males under white light exposure. Due to UV, violet (350–440 nm) and green (500–560 nm) wavelength spectra were often used to trap Cerambycid beetles, therefore, 6 LED light wavelengths, which were UV (365 nm), violet (420 nm and 435 nm), green (515 nm), and red (600 nm and 660 nm), were selected to investigate the effect of exposure time on the phototactic behavioral responses of females and males, thus providing an appropriate test condition for the next experiment. Then, we displayed the phototactic behavioral responses of females and males under different light wavelengths at five time slots to show diel rhythm on the phototactic behavioral response. Finally, at the transcriptome level, we investigated the gene expression profiles of females and males under different light exposure times (15 and 120 min) of white light relative to dark conditions (control treatment) to explore the response mechanism for light exposure. Thus, these results not only can provide valuable information for the monitoring and management of population density and prediction, but also may expand the understanding of the molecular response mechanism in the nocturnal beetle *D. helophoroides* to light exposure.

## 2 Materials and methods

### 2.1 Insects

Adults of the beetle *D. helophoroides* were provided by the Institute of Entomology, College of Agriculture Vocational of Beijing. The first generation of the wild population was collected from parasitized larvae and pupae of *A. glabripennis*. A substitute hosts (*Thyestilla gebleri* Fald.) were used to rear larvae. Adults were reared at 25°C and 60% ± 10% relative humidity under an LD cycle of 16:8 h (light: 500 lx and dark 0 lx) in an artificial climate chamber (RXZ-500D, Jiangnan Instrument Factory, Ningbo, China). The insects were provided water in moist cotton balls placed on the centrifuge tube and were fed *Tenebrio molitor* L. larvae that were dried by the stove at 60 °C. Female and male adults were distinguished by the end angle of the anal plate under a dissecting microscope (Olympus SZ51, Tokyo, Japan) according to Tang et al. (2007). Approximately 30–60 days after emergence adults were used in our experiment, and all females and males were able to mate and oviposit normally (female) in our experiment.

### 2.2 Experimental apparatus and light sources

**FIGURE 1:**
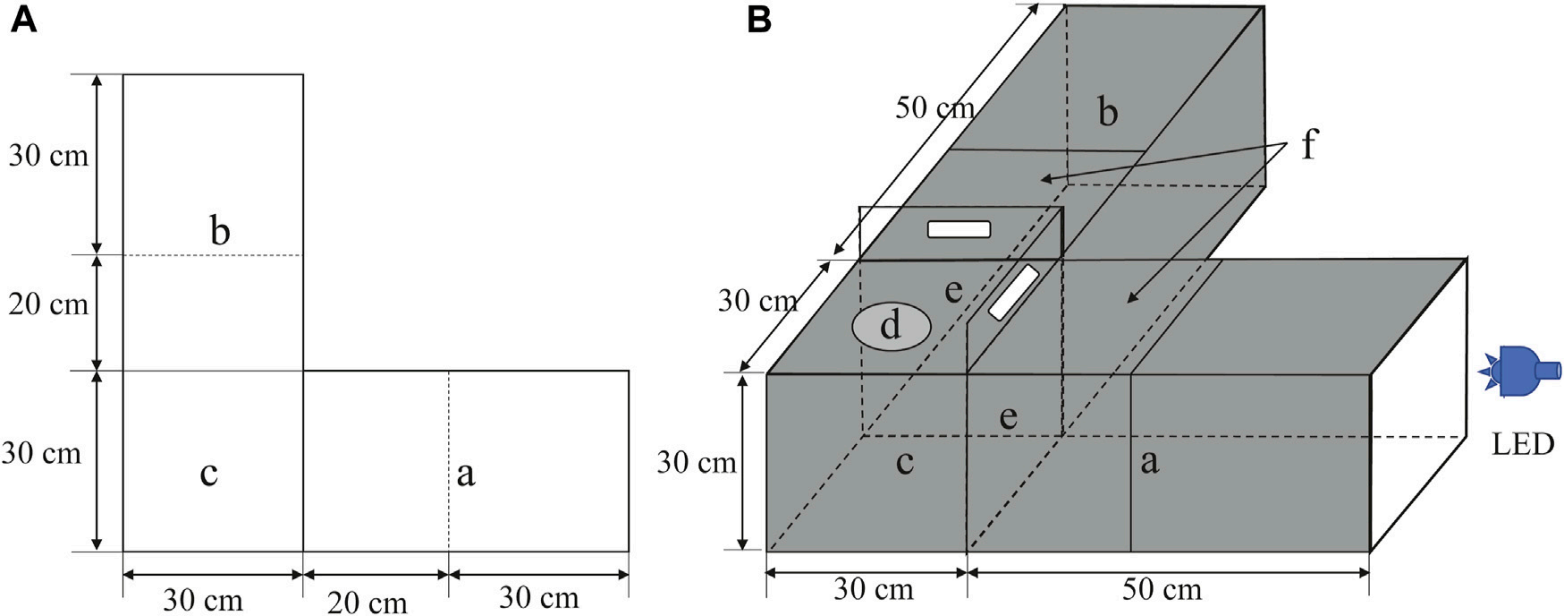
Schematic diagram of the behavioral bioassay chamber. **(A)** planar diagram, **(B)** stereoscopic diagram. Storage chambers (a and b); activity chamber (c); test insect release point (d); black opaque acrylic boards (e) and moveable transparent acrylic board (f). LED: light emitting diode.

To observe the phototactic behavior of *D. helophoroides* in the different conditions, a light-dark alternative apparatus with an opaque black acrylic board was self-designed based on a previous study (Figures 1A, B) (Jiang et al., 2023a). The apparatus comprised two test chambers (L = 50 cm × H = 30 cm × W = 30 cm) at right angles to each other, which provided an activity space for beetles to respond to different wavelength LED lights, and an activity chamber (L = 30 cm × H = 30 cm × W = 30 cm), which provided adequate space for insect movement. The side of the test chamber near the light source area was called the light area (Figure 1B, a), and the other side without the light source area was called the dark area (Figure 1B, b). To conveniently observe the behavior of beetles, transparent acrylic boards were used on the top side of the light and dark areas. Half of the top of the chamber (Figure 1B, f) was used with a moveable transparent acrylic board to facilitate the removal and observation of insects after the test. At the tops of the activity chamber, we drilled a hole with 10 cm diameter (Figure 1, d) to introduce test insects, and then placed a transparent acrylic board on the hole to prevent insect escape when *D. helophoroides* was added to the activity chamber. A transparent and opaque acrylic board was fitted on the end of the light and dark areas to prevent insect escape and facilitate light transmission, respectively. We set up two baffle boards with opaque black acrylic (Figure 1, e) between the test and activity chambers. Only the LED light resource was turned on in the light area when the baffle was pushed down (Figure 1, a). The LED light resource was spread in the light area (Figure 1, a) and activity chamber (Figure 1, c), and there was no light in the dark area (Figure 1, b) when the baffle was removed. The number of test insects in the phototaxis areas within 30 cm was regarded as the positive trapping number of females and males, while the number of insects was used as the negative trapping number of adults in the dark area. To avoid the effect of light reflection, opaque black acrylic boards were used in the outer and inner of chambers, except for the top side of the light and dark areas, to facilitate the observation of test insects. All experiments were tested under a completely dark environment in an air-conditioned room to maintain a constant temperature of 25°C ± 1°C and 65% ± 10% RH.

Fourteen monochromatic LED lamps distributing ultraviolet (UV) and visible light were used as light stimuli at the end of the light area, including 365, 385, 400, 420, 435, 475, 500, 515, 560, 595, 600, 630, and 660 nm (Jiang et al., 2023a). All LED lamps were made by Shenzhen Xinhongxian Electronics Technology Co., Ltd. (Shenzhen, China), and the power of each lamp was 20 W. A rheostat was connected LED lights to adjust the light intensity. Due to females and males were attracted by light passing through a transparent acrylic board rather than under direct light, the light irradiance at the center of the activity chamber was measured by a high-speed spectrometer (HSU–100S, Asahi Spectra Co. Ltd., Tokyo) with sensor fiber. The intensity of the light in photons per second per area was adjusted and maintained at 6.31 × 10^18^ (photos/m^2^/s) (Miyatake et al., 2016).

### 2.3 Behavioral experiments

A series of dual-choice experiments were displayed to determine the effect of exposure time, diel rhythm, and light wavelength on the phototactic behavioral response of female and male *D. helophoroides* in a completely dark indoor environment. Different light sources were installed at the end of the light area, while no light sources were installed at the end of the dark area. Twenty adult females or males were used as one replicate to test the phototactic behavioral response of adults. In each test, adults were placed in the activity chamber to adapt to the dark environment for 2 h, and then the LED lamps were turned on. When the baffles were removed from the junction between the activity and the test chambers, the timer was started. The number of females and males was recorded in the light, dark and release areas, respectively. To avoid residual adult volatile cues, the activity and test chamber was wiped with hexane and air-dried between trials.

#### 2.3.1 Effect of exposure time on phototactic behavioral responses

UV, violet (350–440) and green (500–560) wavelength spectra were often used to trap Cerambycid beetles in field (Kim et al., 2019; Van der Kooi et al., 2021). Red light was also the preferred source of light for adult *D. helophoroides* (Wang et al., 2021). Therefore, six wavelength spectra (365, 420, 435, 515, 600, and 660 nm) were selected to analyze the effect of exposure time on the behavioral responses of adults in the time slot 19:00–21:00 to provide a suitable light exposure time for the next test. We installed 6 different wavelength LED lights at the end of the light area, while no lights were installed at the end of the dark area. The number of beetles in the light area, dark area and release area were recorded at 15, 30, 45, 60, 75, 90, 105, and 120 min during the observation period, respectively. Twelve replicates were measured at each light wavelength.

#### 2.3.2 Monochromatic LED light preference in different time slots

To determine the effect of different wavelengths on the phototactic behavioral responses of female and male *D. helophoroides*, a series of experiments were conducted in 5 different time slots (9:00–11:00, 14:00–16:00, 19:00–21:00, 0:00–2:00, and 5:00–7:00). The female or male beetles either crawled or flew into the light or dark area from the activity chamber. The number of beetles in the light area, dark area and release area was recorded after an illumination time of 15 min based on the results of “Effect of exposure time on the phototactic behavioral responses” test. The samples of females and males were 6 replicates per time slot, and thirty replicates at five time slots were measured for 14 wavelengths of light (365–660 nm).

### 2.4 Transcription analysis

#### 2.4.1 Transcriptome sequencing, assembly, and functional annotation

Approximately 500 (female and male) heads of adult *D. helophoroides* were used for RNA extraction in the 6 different treatments, including dark adaptation of 120 min, light exposure time of 15 and 120 min after 120 min dark adaptation (white light intensity: 6.31 × 10^18^ (photos/m^2^/s)). Total head RNA was extracted using TRIzol Reagent (Invitrogen, Waltham, MA, United States) based on the manufacturer’s standard protocol. Then, the extracted RNA was determined using a 5,300 Bioanalyser (Agilent) and quantified using an ND-2000 (NanoDrop Technologies), respectively. Only high-quality RNA samples (OD260/280 = 1.8–2.2, OD260/230 ≥ 2.0, RIN≥6.5, 28S:18S ≥ 1.0, >1 μg) were used to construct the sequencing library. The total head RNA samples were sent to Shanghai Majorbio Bio-pharm Biotechnology Co., Ltd. (Shanghai, China) for RNA quality testing, library construction, and sequencing at a depth of 6.84 G according to the manufacturer’s instructions (Illumina, San Diego, CA). All data have been uploaded in the NCBI Sequence Read Archive (SRA) database (http://www.ncbi.nlm.nih.gov/bioproject/987166). Afterward, the clean data were mapped to the reference genome of *D. helophoroides* using HISAT2 software (Zhang et al., 2023). All mapped genes were aligned into public databases using Diamond (v0.8.22, E-value 1e-5), including the NCBI non-redundant nucleotide sequences (NR), Swiss-Prot, gene ontology (GO), Kyoto Encyclopedia of Genes and Genomes (KEGG), clusters of orthologous groups of proteins (KOG/COG/eggNOG), and protein family (Pfam) databases as described previously (Liu et al., 2023).

#### 2.4.2 Screening of DEGs, GO and KEGG enrichment analysis

The expression level of genes was calculated according to the fragments per kilobase of exon model per million mapping read values (FPKM) method to identify differentially expressed genes (DEGs) between two different samples. Differential expression analysis of different treatments was displayed using DESeq2 (http://bioconductor.org/packages/stats/bioc/DESeq2/) according to an absolute value of Log_2_ FC (fold change) ≥ 1.0 and a false discovery rate (FDR) < 0.05. Furthermore, GO and KEGG functional enrichment analyses were performed to identify which DEGs were significantly enriched in GO terms and KEGG pathways at Benjamini–Hochberg (BH) - corrected FDR <0.05 compared with the whole-transcriptome background. Goatools and KOBAS were used to determine GO functional enrichment and KEGG pathways, respectively (Xie et al., 2011).

#### 2.4.3 Short time-series expression miner (STEM) analysis

STEM analysis was used to investigate the dynamic expression of DEGs in beetles in response to light exposure (Ernst and Bar-Joseph, 2006). In the STEM analysis, the light exposure time and the calculated Log2 value of the ratio of their mRNA levels (in FPKM) at light exposure of 15 or 120 min to the control (dark adaptation = 0 min) were used as the horizontal and vertical coordinates. Subsequently, total DEGs were clustered into distinct and significant temporal expression clusters using default settings according to previous studies described (Zheng et al., 2020; Wang Z. et al., 2023).

#### 2.4.4 WGCNA and gene-gene interaction analyses

An unsigned topological overlap matrix (TOM) was used to construct the WGCNA network according to the following parameters: the network type was signed, soft power was 14, min module size was 30, minKMEtoStay was 0.3, and merge cut height was 0.25 (Wang Z. et al., 2023; Liu et al., 2023). A correlation heatmap was constructed between the modules and traits after the cluster analysis of the modules. The module eigengene value was calculated to determine the association of modules with each light exposure time for 18 samples. The STRING database (v11.5, https://cn.string-db.org/) was used to analyze GGI predictions of hub genes, and Cytoscape software (v3.9.1) was used to visualize the interaction network from STRING (Shannon et al., 2003; Szklarczyk et al., 2020).

#### 2.4.5 Quantitative real-time PCR (qRT-PCR) analysis

The DEGs from the transcriptome data were randomly selected to confirm the RNA-seq data and identify their transcript levels using qRT-PCR method. We used Primer Premier 5.0 software to design the primers based on the selected transcript sequence, and then these selected genes were amplified (Supplementary Table S1). We used a CFX96 Touch Deep Well Real-Time PCR Detection System (Bio-Rad, Hercules, CA, United States) to perform qRT-PCR test. The relative expression levels of mRNA were calculated with the GAPDH gene, which is an internal control gene, using the 2 ^−ΔΔCT^ method (Livak and Schmittgen, 2001). Three biological replicates were measured for each treatment.

### 2.5 Statistical analysis

To evaluate the trapping ability of different light sources for female and male *D. helophoroides* at different treatments, the trapping rate was used as an important parameter to evaluate the trapping ability of LED lights.

The trapping rate was calculated using the following formula:

$$Trapping\;rate\;\left(\%\right)=\frac{Number\;of\;beetles\;in\;light\;area}{Total\;number\;of\;test\;beetles}\times 100\;$$ (1)

Furthermore, in “Monochromatic LED light preference” experiment, the preference index (PI) was also used as an important parameter to evaluate the preference of beetles for light wavelengths. The formula is as follows:

$$\text{PI }\left(\%\right)=\frac{Number\;of\;beetles\;in\;light\;area-Number\;of\;beetles\;in\;dark\;area}{Number\;of\;beetles\;in\;light\;area+Number\;of\;beetles\;in\;dark\;area}$$ (2)

The effects of light exposure time, diel rhythm, and light wavelength on the phototactic response of female and male beetles on trapping rate were analyzed using Generalize linear model (GLM) with a Poisson distribution and a log link function and followed by the Bonferroni test. Non-responders were recorded but excluded from the analysis. All experimental data were statistically analyzed using SPSS Statistics v. 21.0 (IBM Corp., Armonk, NY, USA) for Windows.

## 3 Results

### 3.1 Phototactic behavioral response of male and female *D. helophoroides*

#### 3.1.1 Effect of exposure time on the phototactic behavioral response

**FIGURE 2:**
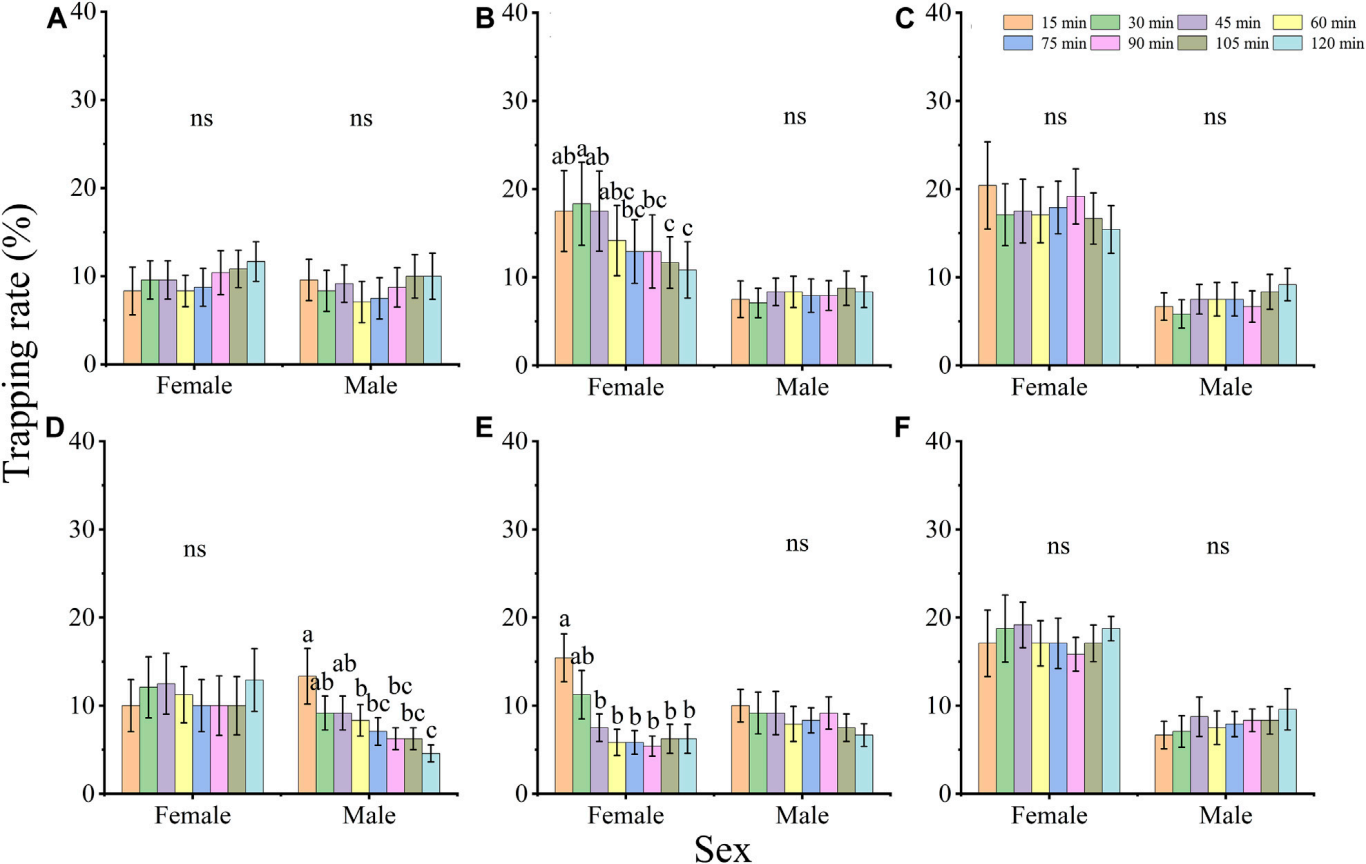
The effect of exposure time on the phototactic behavioral response rate of adults *D. helophorides* at wavelengths of 365 nm **(A)**, 420 nm **(B)**, 435 nm **(C)**, 515 nm **(D)**, 600 nm **(E)** and 660 nm **(F)**. The significant differences in phototactic behavioral responses to LED lights among different exposure times were analyzed using GLM with Poisson distribution and a log link function followed by Bonferroni test; α = 0.05. The samples of females and males were 12 replicates. The data are presented as the mean ± SE.

The trapping rates of females and males showed different changes under the 6 LED light wavelengths (Figure 2). The trapping rate of females at 420 and 600 nm, and that of males at 515 nm decreased gradually when the exposure time was prolonged (female, 420 nm: *X* ^2^ = 48.000, *df* = 7, *p* < 0.001, 600 nm: *X* ^2^ = 88.085, *df* = 7, *p* < 0.001; male, 515 nm: *X* ^2^ = 67.051, *df* = 7, *p* < 0.001); and the trapping rate of adults at light exposure 15 min was higher than at 120 min. The trapping rates of females and males show no significant difference among the different exposure times under the other wavelengths (females, 365 nm: *X* ^2^ = 12.441, *p* = 0.087, 435 nm: *X* ^2^ = 10.968, *p* = 0.140, 515 nm: *X* ^2^ = 11.599, *p* = 0.115, 660 nm: *X* ^2^ = 6.324, *p* = 0.502; males, 365 nm: *X* ^2^ = 12.240, *p* = 0.093, 420 nm: *X* ^2^ = 3.094, *p* = 0.876, 435 nm: *X* ^2^ = 12.148, *p* = 0.096, 600 nm: *X* ^2^ = 12.427, *p* = 0.087, 660 nm: *X* ^2^ = 9.197, *p* = 0.239; all *df* = 7).

#### 3.1.2 Monochromatic LED light preference in different time slots

**FIGURE 3:**
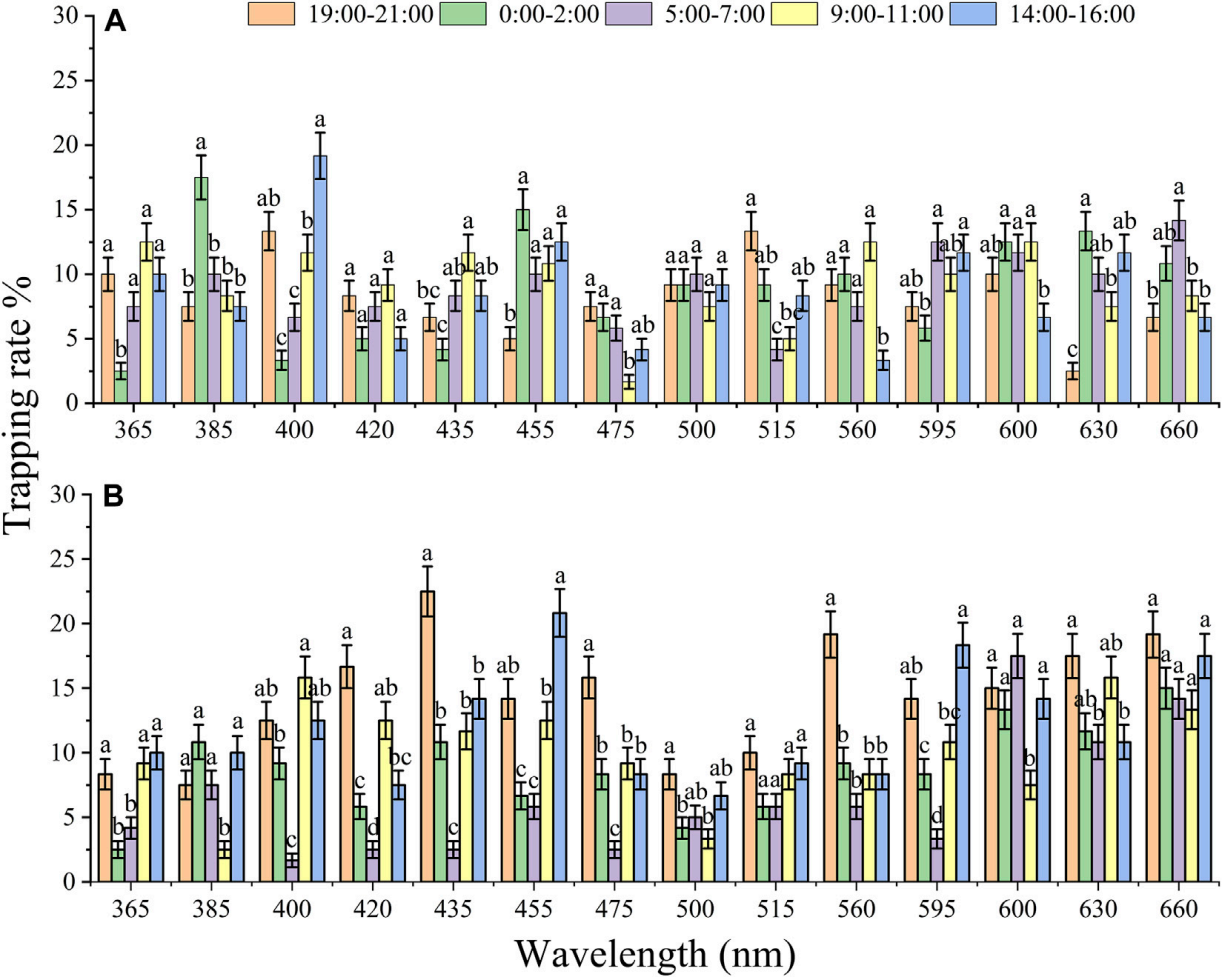
The effect of diel rhythm on the phototactic response rate of males **(A)** and females **(B)**. The significant difference was analyzed by GLM with Poisson distribution and a log link function followed by Bonferroni test; α = 0.05, and the same lowercase letters on top of the bar indicate no significant difference under the same wavelength. The samples of females and males were 6 replicates per time slots. The data are presented as the mean ± SE.

To compare the effect of diel rhythm on phototactic behavioral responses, the trapping rates of females and males were compared at five different time slots (Figure 3). The behavioral responses of females and males showed a significantly difference among 5 time slots, except for those of males under 500 nm (*X* ^2^ = 2.432, *df* = 4, *p* = 0.657) and females under 660 nm (*X* ^2^ = 8.541, *df* = 4, *p* = 0.074). Under 515 and 630 nm illumination, both males and females exhibited higher preference at night (19:00–2:00) than at other time slots, but the highest trapping rates of males and females were 13.33% and 10.00% (515 nm) and 13.33% and 17.50% (630 nm), respectively (Figures 3A, B; 515 nm, male: *X* ^2^ = 38.412, *df* = 4, *p* < 0.001, female: *X* ^2^ = 11.768, *df* = 4, *p* = 0.019; 630 nm, male: *X* ^2^ = 81.094, *df* = 4, *p* < 0.001, female: *X* ^2^ = 16.005, *df* = 4, *p* = 0.003). Moreover, the trapping rates of males at 385 and 455 nm and females at 420, 435, 475, and 560 nm showed higher preference at night (19:00–2:00) than during the day (Figure 3), while those of males at 400 nm, and females at 455 nm and 595 nm showed higher phototactic responses during the day (14:00–16:00) (Figure 3).

**FIGURE 4:**
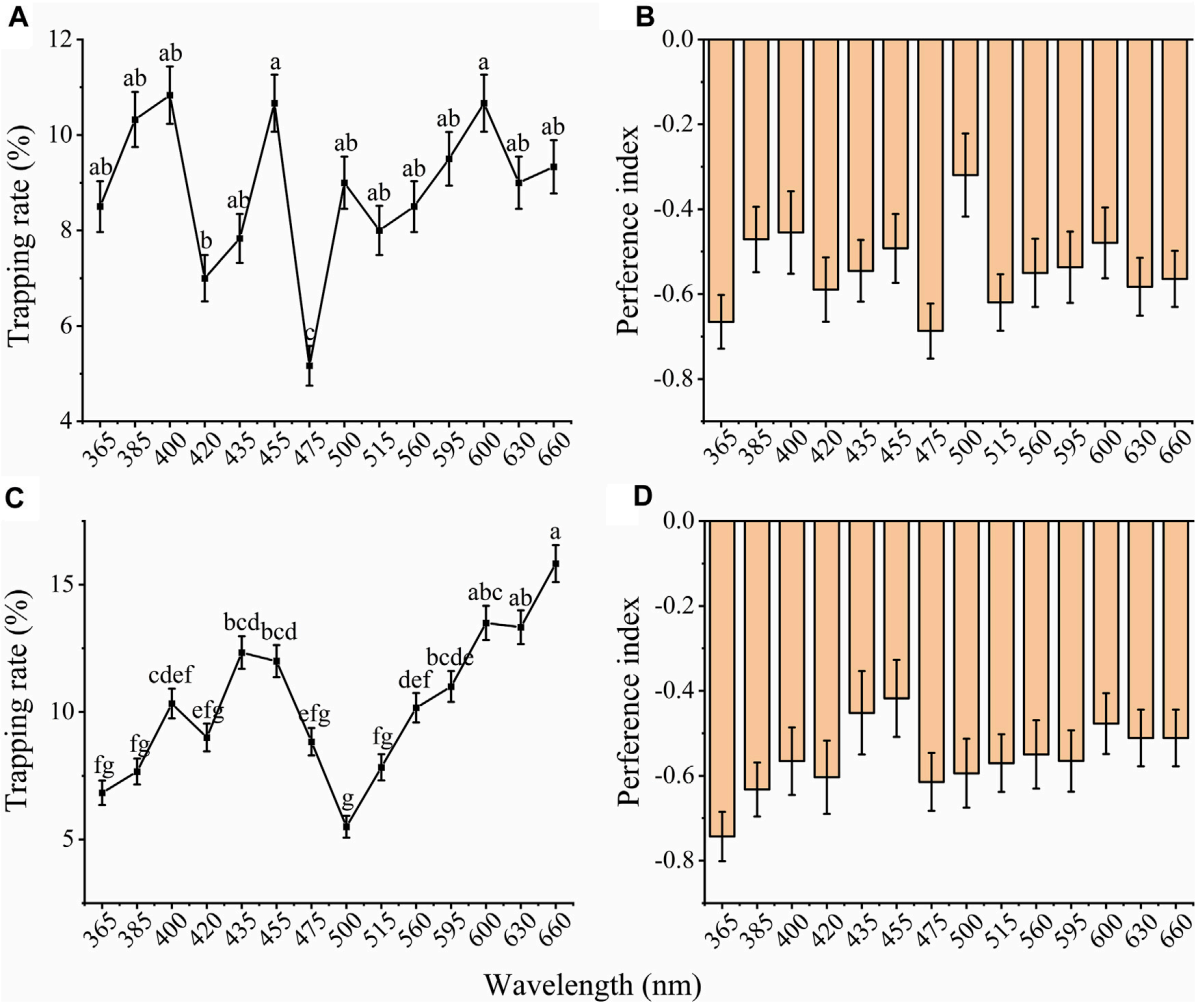
Total trapping rates and preference index of males and females to different wavelengths of light. The total trapping rate of males **(A)** and females **(C)**. The preference index of males **(B)** and females **(D)** to different wavelengths of light. The significant difference was analyzed by GLM with Poisson distribution and a log link function followed by Bonferroni test; and the same lowercase letters on top of the line indicate no significant difference. The samples of females and males were 30 replicates per wavelengths. The data are presented as the mean ± SE.

Furthermore, both females and males showed a significant difference among the phototactic behavioral responses of adults under 14 different wavelength LED cues (Figure 4 A and C; male: *X* ^2^ = 164.338, *df* = 13, *p* < 0.001; female: *X* ^2^ = 333.859, *df* = 13, *p* < 0.001). Males and females exhibited different preferences under 14 different LED lamps. Males showed the highest preference for 400, 455, and 600 nm, followed by 385 and 660 nm (Figure 4A), while females exhibited the highest preference at 660 nm, followed by 600, 630, 435 and 455 nm (Figure 4C). The trapping rates of males were 10.83%, 10.67% and 10.67% under 400, 455, and 600 nm LED lights, respectively (Figure 4A), while the trapping rate of females was 15.83% under 660 nm (Figure 4C). The lowest trapping rate of males and females was in the blue-green region (male: 475 nm, 5.17%, female: 500 nm, 5.50%). The preference index of females and males showed that the beetles had distinct negative phototaxis under illumination with different wavelengths (Figures 4B, D).

### 3.2 Head transcriptome sequencing of *D. helophoroides*

#### 3.2.1 RNA sequence analysis

**FIGURE 5:**
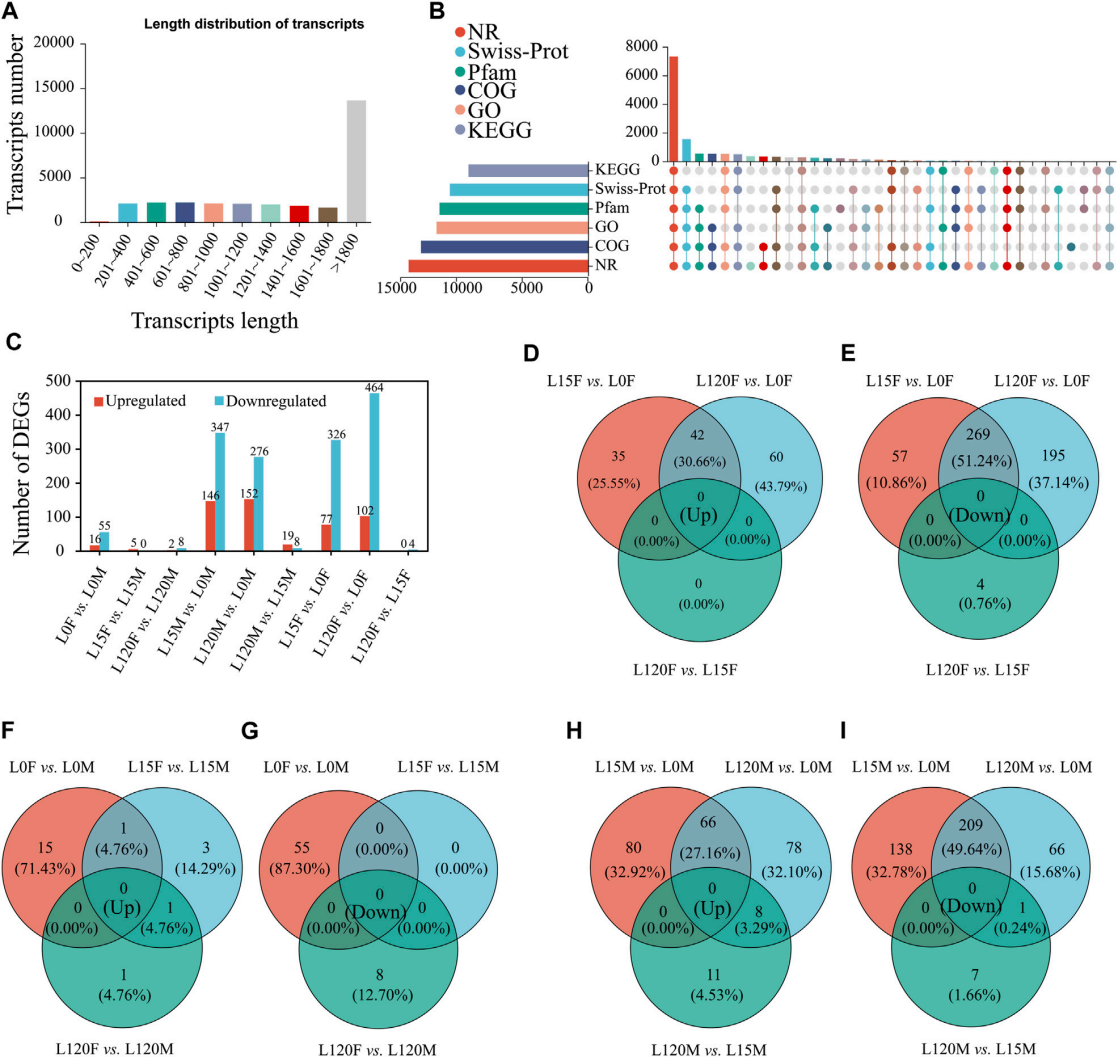
Illumina sequencing of the heads of adults *D. helophorides* under the different light exposure times. **(A)** The length distribution of all assembled transcripts. **(B)** The number of genes annotated via different databases, including NCBI non-redundant protein database (NR), NCBI non-redundant nucleotide database (NT), Swiss-Prot, gene ontology (GO), Kyoto Encyclopedia of Genes and Genomes (KEGG), clusters of orthologous groups of proteins (KOG), and protein family (Pfam). **(C)** Classification information of annotated GO terms. **(C)** the number of DEGs in the different treatments. The Venn diagram of upregulated and downregulated genes in the comparisons of females **(D,E)**, females and males **(F,G)**, and males **(H,I)**.

To further investigate the underlying molecular mechanisms of light signal recognition of *D. helophoroides*, we performed a time-course transcriptome analysis using head samples of female and male beetles exposed to white light for different lengths of time (0, 15, or 120 min). Eighteen samples were subjected to RNA-Seq, which produced approximately 140.96 G (6.84 Gb of data for each sample). Quality scores were more than 98.04% and 94.08% at the levels of Q20 and Q30, respectively (Supplementary Table S2). Subsequently, the clean reads of each sample were mapped to the reference genome, and the mapping rate ranged from 88.08% to 90.41% (Supplementary Table S3). A total of 14,890 genes were mapped, and 2,164 new genes were found and generated 29,851 transcripts in these transcriptome sequences. There were 13,647 transcripts (45.7%) with lengths ≥1,800 bp, 7,525 transcripts (25.2%) in the range 1,000–1,800 bp, and 8,679 (29.1%) with lengths ≤1,000 bp in our transcriptome datasets (Figure 5A). Among 14,890 genes, 92.01% (13,700 genes) were annotated via the NR database, 86.00% (12,805 genes) were annotated via the COG database, 77.71% (11,571 genes) were annotated via the GO database, 75.57% (11,252 genes) were annotated via the Pfam database, 70.79% (10,541 genes) were annotated via the Swiss-Prot database, and 62.00% (12,805 genes) were annotated via the KEGG database (Figure 5B).

#### 3.2.2 Identification of differentially expressed genes (DEGs)

Differentially expressed genes were identified using DESeq 2. A total of 1,052 DEGs were identified in *D. helophoroides* via pairwise comparisons, among which 403, 566, 493, and 428 DEGs were obtained in the four comparisons of dark adaptation and light adaptation after light exposure for 15 and 120 min (L15F vs. L0F, L120F vs. L0F, L15M vs. L0M, L120M vs. L0M), respectively (Figure 5C). Furthermore, the results showed that there were very few genes in the common upregulated and downregulated comparison of males with females under the same light exposure times (Figures 5F, G), while the genes were relatively lot up- or downregulated uniquely in the comparisons between L15F vs. L0F, L120F vs. L0F, L15M vs. L0M, and L120M vs. L0M (Figures 5D, E, H, I).

#### 3.2.3 Functional classification of DEG responses to light and dark adaptation

**FIGURE 6:**
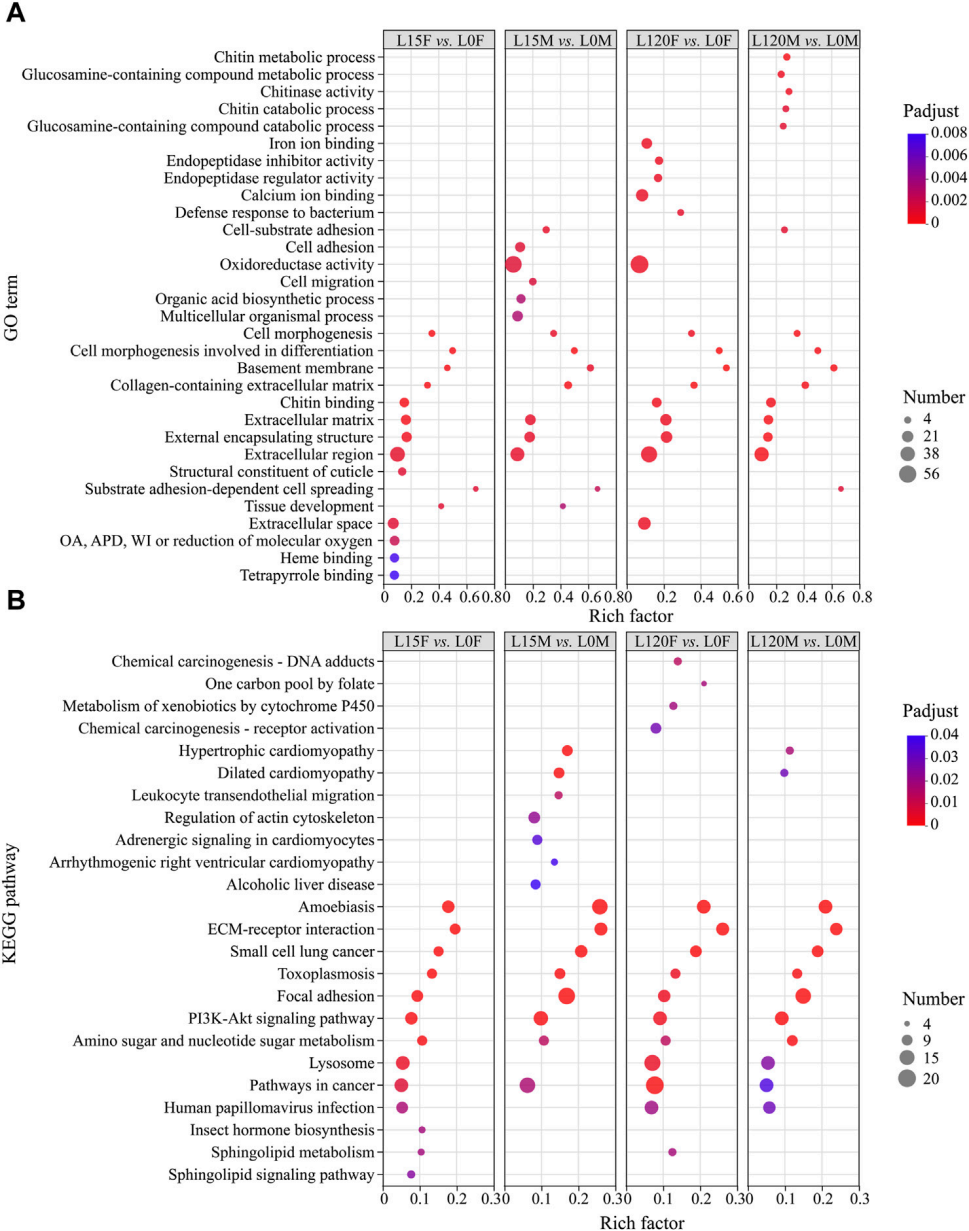
The gene Ontology (GO) term **(A)** and Kyoto Encyclopedia of Genes and Genomes (KEGG) pathway **(B)** enrichment analysis of all DEGs in the different comparisons. OA, APD, oxidoreductase activity, acting on paired donors, with incorporation or reduction of molecular oxygen.

To reveal the effect of light treatment on the transcriptomes of females and males relative to dark treatment, GO enrichment analysis was used to classify the DEGs (Figure 6A). The GO terms of both females and males were markedly enriched in “cell morphogenesis,” “cell morphogenesis involved in differentiation” (biological process), “basement membrane”, “extracellular region,” “collagen-containing extracellular matrix,” “extracellular matrix,” “external encapsulating structure” (cellular component), and “chitin binding” (molecular function) (Figure 6A).

To further display the biological function of DEGs, the DEGs were analyzed using KEGG pathway analysis based on the KEGG database. The top pathways were displayed for the prominent enriched DEGs between light exposure and dark conditions (Figure 6B). In the L120F vs. L0F and L15F vs. L0F comparisons in female, 242 and 155 DEGs were annotated to 190 and 186 KEGG pathways, respectively (Supplementary Tables S4, S5), while in the L120M vs. L0M, L15M vs. L0M comparisons in males, 190 and 238 DEGs were annotated to 215 and 242 KEGG pathways, respectively (Supplementary Tables S6, S7). These DEGs were significantly enriched in “ECM-receptor interaction,” “Focal adhesion,” “PI3K-Akt signaling pathway,” and “Lysosome” (Figure 6B).

#### 3.2.4 STEM analysis of the DEGs in the response to light exposure

**FIGURE 7:**
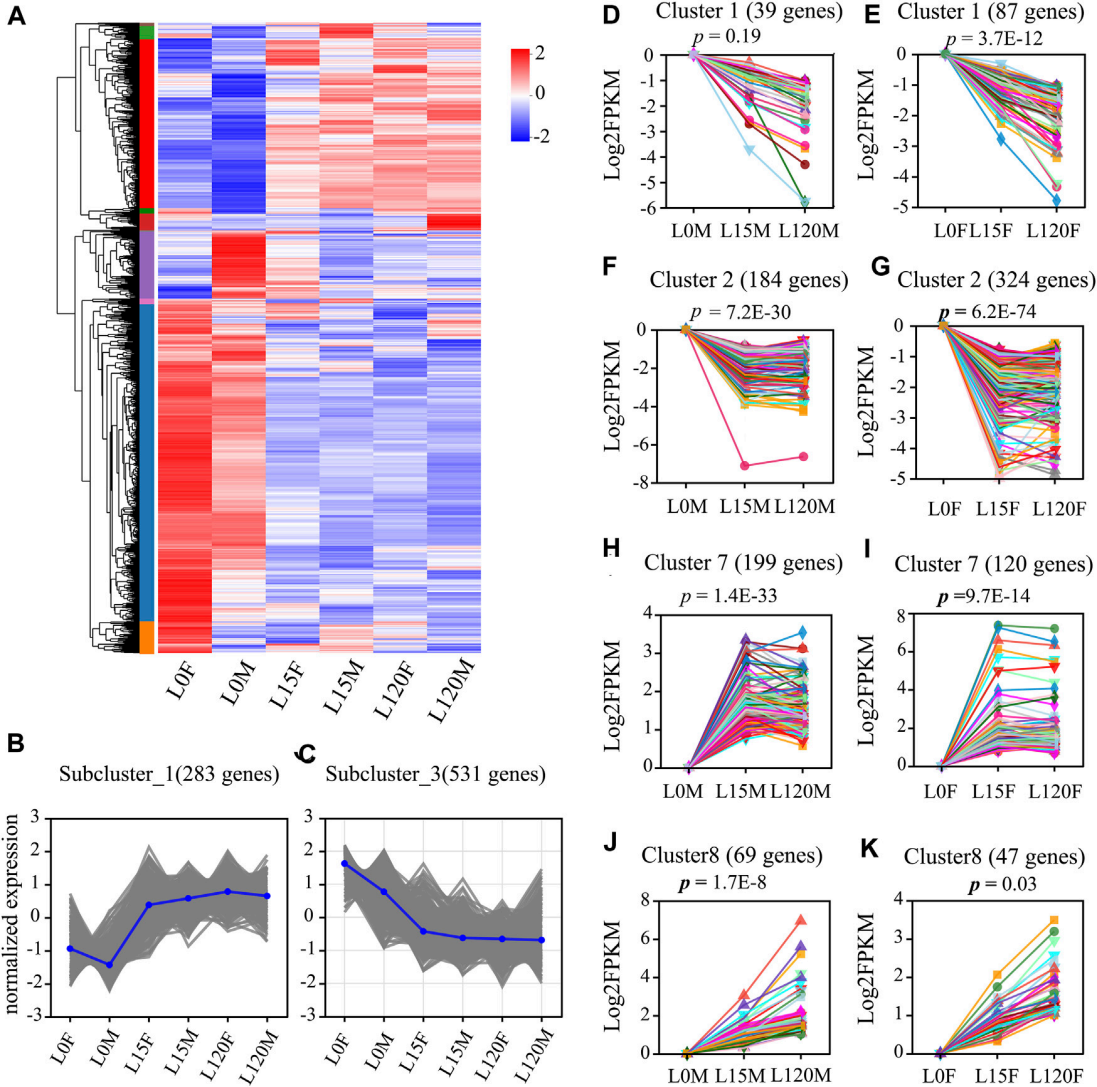
Summary of differentially expressed genes (DEGs) in the head of *D. helophorides* under the different light exposure times. **(A)** Expression profiles of the DEGs at light exposure time points of female and male (0, 15, 120 min) using a heatmap. **(B,C)** Two of the subclusters were showed. **(D–K)** Temporal expression analysis of the DEGs in the head of *D. helophorides* following light exposure at 0, 15 and 120 min using STEM software (male: D, F, H, and J; female: **(E,G,I,K)**. There data from 15 to 120 min were normalized using log2 compared to dark adaption (light exposure 0 min).

To further explore the expression patterns of these DEGs, hierarchical clustering was performed on their expression profiles at different treatment times. The results showed that the DEGs were divided into two groups, including dark treatment (L0F and L0M) and light treatment (L15F, L120F, L15M, and L120M) (Figure 7A). Sub-clustering analysis showed that the relative expression of DEGs changed pattern with prolonged light exposure time (Figures 7B, C).

Furthermore, STEM analysis was performed to further explore the expression patterns of these DEGs at different treatment times in females and males (Figures 7D–K). The DEGs were clustered into eight distinct temporal expression patterns in females and males, and three clusters in the male comparison and four clusters in the female comparison showed significant change patterns following light exposure time (Figures 7D–K, *p* < 0.03). The other cluster showed no significant change patterns (Supplementary Figures S2A–H). The predominant profiles revealed that the expression patterns of most DEGs were rapidly induced within the first 15 min of light exposure (Figures 7D–K). However, there was no significant difference in the expression patterns of DEGs between light exposure for 15 and 120 min.

#### 3.2.5 WGCNA analysis of the DEGs in the response to light exposure

**FIGURE 8:**
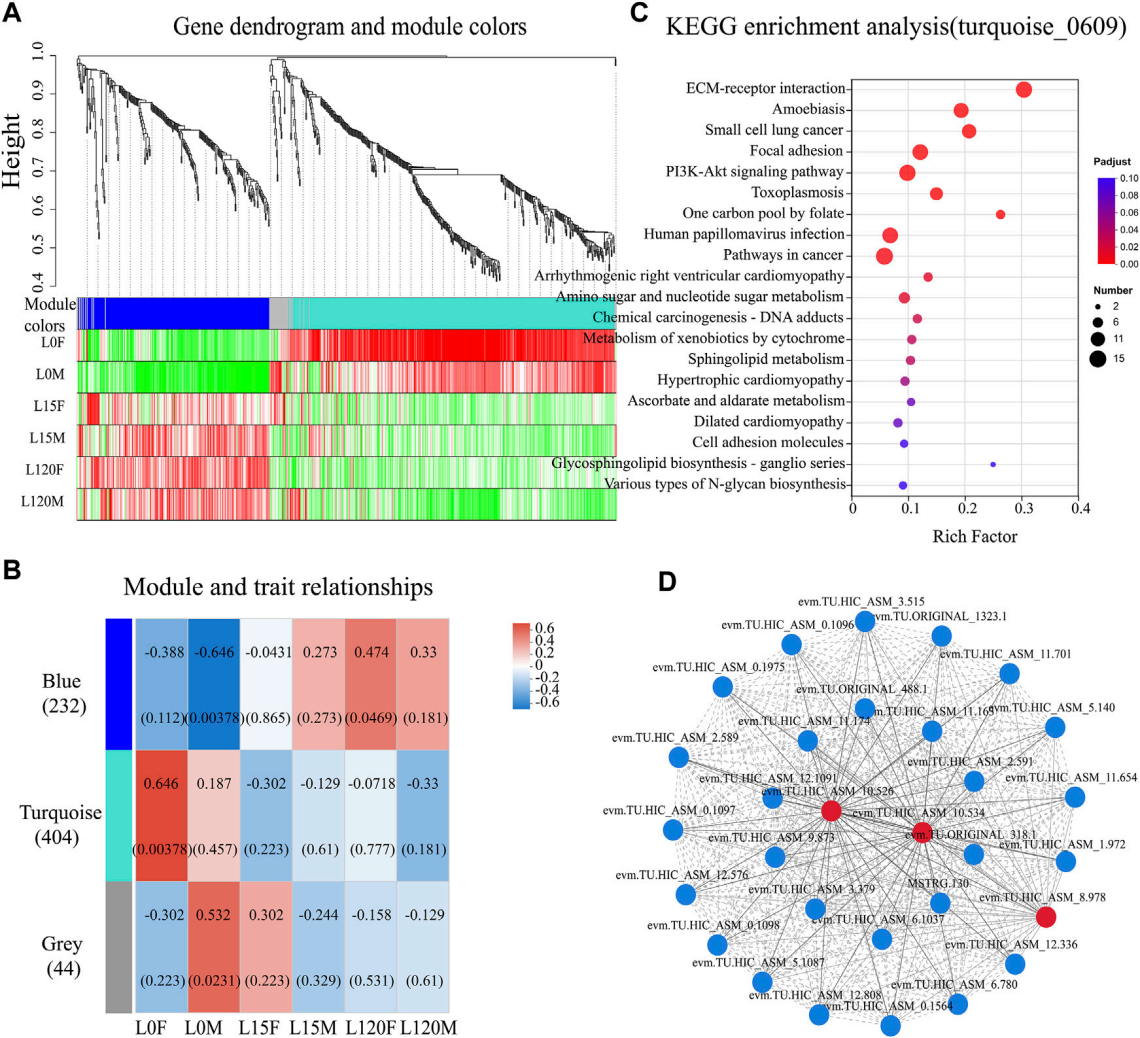
Weighted gene co-expression network analysis (WGCNA), KEGG and gene-gene interaction (GGI) analysis for differentially expressed genes (DEGs) in the head transcriptome data. **(A)** the 1,052 DEGs were clustered three co-expression modules using WGCNA analysis. **(B)** Correlation between module and trait. A correlation analysis between the module and different light exposure times (0, 15, and 120 min) of female and male. **(C)** The genes in the turquoise module were subjected to KEGG enrichment analysis. The top 20 enriched KEGG terms were displayed. **(D)** GGI networks of the DEGs belonging to “ECM-receptor interaction,” “Focal adhesion,” “PI3K-Akt signaling pathway,” and “Lysosome” (Downregulated genes).

A total of 1,052 was performed to further explore key gene co-expression modules in the head of *D. helophoroides* in response to light exposure at different times (0, 15, 120 min) (Supplementary Table S8). Three different co-expression modules were identified to mark different colors based on the similarity in the expression of DEGs (Figure 8A). DEGs in the same co-expression module showed distinct expression profiles in the head of *D. helophoroides* at different light exposure times, especially the blue and turquoise modules (Figure 8B). The blue module of 232 genes had the strongest negative correlation with dark adaptation in males (R = −0.646, *p* = 0.00378) and a weak correlation with light exposure for 120 min in females (R = 0.474, *p* = 0.0469), while the turquoise module of 404 genes had the strongest positive correlation with dark adaptation in female (R = 0.646, *p* = 0.00378). The grey module of 44 genes had the strongest correlation with dark adaptation in males (R = 0.532, *p* = 0.0231) (Figure 8B).

To identify the hub genes in the module, the key genes with a high connectively degree were screened in the co-expression network modules. In the blue modules, the genes (evm.TU.HIC_ASM_8.152) were involved in phosphatidylinositol binding, (evm.TU.HIC_ASM_7.1150) belonged to the integral component of membrane, and (evm.TU.HIC_ASM_10.101) were responsible for sensory perception of light stimulus (Supplementary Table S9). In the turquoise, (evm.TU.HIC_ASM_2.289) was involved in the integral component of the membrane, (evm.TU.HIC_ASM_9.491) was related to flavin adenine dinucleotide binding, and (evm.TU.HIC_ASM_4.1260) and (evm.TU.HIC_AS-M_8.362) encoded calcium ion binding protein (Supplementary Table S10). In the grey modules, (evm.TU.HIC_ASM_8.361) participated in calcium channel activity, and (evm.TU.H-IC_ASM_4.921) was related to transmembrane transporter activity (Supplementary Table S11).

All genes from three modules were subjected to KEGG enrichment analysis, showed that the turquoise module genes were significantly enriched in “ECM-receptor interaction,” “Focal adhesion” and “PI3K-Akt signaling pathway” (*p* <0.001) (Figure 8C). Additionally, the pathways “Amino sugar and nucleotide sugar metabolism,” “Metabolism of xenobiotics by cytochrome P450,” and “Sphingolipid metabolism” also displayed an adjusted *p*-value <0.05. These results suggest that these KEGG pathways may have an important role in the response of *D. helophoroides* to light exposure, especially those pathways with *p* < 0.001 (Figure 8C). Furthermore, to our surprise, human disease pathways were enriched significantly, such as “Small cell lung cancer,” “Arrhythmogenic right ventricular cardiomyopathy” (*p* < 0.01). Furthermore, blue module gene were enriched significantly in “Caffeine metabolism” (Supplementary Table S13, *p* < 0.01), the other KEGG pathway were no significantly enriched (Supplementary Tables S13, S14, *p* > 0.05).

As mentioned above, the “ECM-receptor interaction,” “Focal adhesion” and “PI3K-Akt signaling pathway” pathways, which had adjusted *p* values of 2.59 E−11, 8.67E-06, and 3.17E-05, respectively, were ranked number one, four, and five in all downregulated KEGG pathways following light exposure (Figure 8C; Supplementary Table S12). Furthermore, “Lysosome” was also enriched in the comparison L15F vs. L0F, L120F vs. L0F, and L120M vs. L0M (Figure 6B, *p* < 0.05). Therefore, total 30 DEGs from four pathway-related were further analyzed using GGI networks (Figure 8D). As shown in Figure 8D, we detected 30 DEGs in four pathways interacting with each other, such as cell migration, cell morphogenesis involved in differentiation (evm.TU.HIC_ASM_8.978), (evm.TU.HIC_ASM_0.1096, 1,097, 1,098), (MSTRG.130), and (evm.TU.ORIGINAL_488.1); basement membrane (evm.TU.HIC_ASM_10.534), (evm.TU.HIC_ASM_10.526); integrin-mediated signaling pathway (evm.TU.HIC_ASM_11.701), (evm.TU.HIC_ASM_12.1091) (Supplementary Table S15). Notably, cell migration and cell morphogenesis involved in differentiation (evm.TU.HIC_AS-M_8.978) were downregulated by 2.03–2.78 times following light exposure (Supplementary Table S16); the basement membrane (evm.TU.HIC_ASM_10.534), and (evm.TU.HIC_ASM_10.526) were downregulated by 2.33–2.96 times following light exposure (Supplementary Table S16).

#### 3.2.6 Validation of differentially expressed genes by qPCR

**FIGURE 9:**
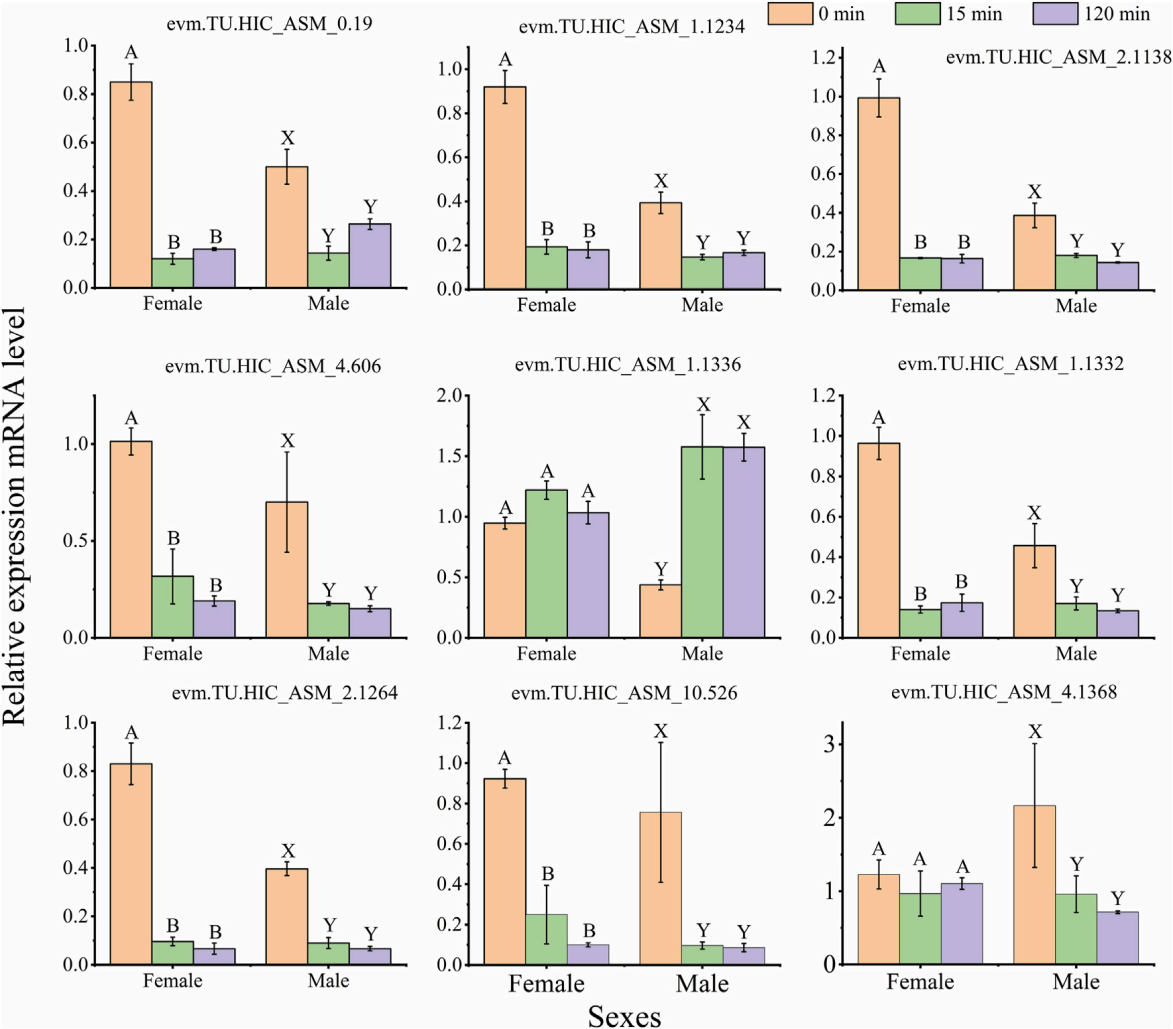
Quantitative PCR (qPCR) data for the mRNA expression profiles of differentially expressed genes (DEGs) in the female and male. The same capital letters on top of the bar indicate no significant difference (Tukey’s test, α = 0.05; Female: A, B, C; Male: X, Y, Z). The data indicates mean ± standard deviation (SD) (n = 3 biological replicates).

To validate the expression patterns of DEGs in the RNA-seq results, qRT-PCR was selected to quantify 9 genes, including basement membrane (evm.TU.HIC_ASM_10.526), The experimental results showed that the expression levels of genes were similar to the results from RNA-seq (Figure 7A; Figure 9, and Supplementary Table S8). Such as, the expression levels of (evm.TU.HIC_ASM_10.526, evm.TU.HIC_ASM_2.1264) were downregulated under light exposure with 15 min, while there was no significant difference between light exposure for 15 and 120 min.

## 4 Discussion

To decrease negative effect of chemical pesticides on the environment, the taxis of insects to artificial lights are widely used to detect, monitor and manage insect pests (Kim et al., 2019; Van der Kooi et al., 2021), such as Tenebrionidae, Curculionidae, Pselaphidae, Silvanidae, Cerambycidae and Scolytinae (Park and Lee, 2017; Marchioro et al., 2020). However, traps with artificial lights can also attract many natural enemies when they are used to detect and manage insect pests (Luo and Chen, 2016; Kim et al., 2019). Therefore, the trapping ability of lights for natural enemies of pests should also be considered in the monitoring and detection of insect pests (Bian et al., 2018). The ectoparasitoid beetle *D. helophoroides* is an important natural enemy of wood-boring insects and is widely used in controlling cerambycid beetles, including *A. glabripennis* and *M. alternatus* (Wang L. et al., 2023; Lyu et al., 2023; Zhang et al., 2023). Therefore, it is vital to explore the response mechanisms of ectoparasitoid beetles to light cue cognition at behavioral and molecular level. Our results showed that female and male *D. helophoroides* have negative or weak phototaxis at a certain light intensity, and it was influenced by light exposure time, diel rhythm, sex, and light wavelength.

Previous studies have shown that *D. helophoroides* adults are attracted by NIR light with a peak wavelength of 700 nm and an intensity at 7 lux, and the highest phototactic response rate is approximately more than 60% in the near-infrared light region (Wang et al., 2021). In the present study, males showed the highest preference for 400, 455, and 600 nm (Figure 4A), while females exhibited the highest preference at 660 nm (Figure 4C). The trapping rates were approximately 10.67%–15.83%. We speculate that the light intensity may cause a significant difference between the two studies. In previous study showed that phototactic behavioral rate of adults decreased with increasing light intensity (Wang et al., 2021). In this study, a 6.31 × 10^18^ (photos/m^2^/s) light intensity (approximately 180 lx) was used, which may also contribute to the low trapping rates of adults.

The trapping rate of females and males was also affected by light exposure time. For example, the trapping rate of females at 420 and 600 nm and males at 515 nm under the light exposure with 15 min was higher than under 120 min (Figures 2B, D, E), so the samples were selected in the light exposure time with 0, 15 and 120 min after dark adaptation in transcriptomic analysis. Moreover, the trapping rate was not significantly different between 15 and 120 min (Figures 2A, C, F). In transcriptomic and qRT-PCR data, there was not significant difference in the majority of gene expression (Figure 7A; Figure 9). Therefore, the treatment sample time interval should be extended to response monochromatic light radiation in the further study.

Previous studies have shown that Coleoptera beetles display a preference for UV, violet and green wavelength spectra (350–440 and 500–560 nm) (Kim et al., 2019; Van der Kooi et al., 2021). Using the same behavioral response chamber, both females and males *A. glabripennis* displayed higher phototactic responses under illumination with 420 and 435 nm wavelengths (violet and blue) at night, with an approximately 74%–82% trapping rate (Jiang et al., 2023a). UV light traps were more attractive to *Arhopalus ferus* and *Prionoplus reticularis* (longhorn beetles) than their adjacent two yellow lights (Pawson and Watt, 2009). UV-blue light traps captured 2–4 times more bark beetles *Hylurgus ligniperda* and *Hylastes ater* beetles than traps baited with other wavelengths in New Zealand (Pawson et al., 2009). These results suggested that UV and blue light traps can be used to detect and control wood boring pests, including longhorn and bark beetles. In this study, females showed a higher preference for 420, 435, 475, 560, and 630 nm at night from 19:00–21:00 (Figure 3B), but the trapping rate decreased with increasing light intensity (Wang et al., 2021). Therefore, light intensity should be enhanced to reduce the trapping of *D. helophoroides* when UV and blue light traps are used to detect and manage wood boring pests.

Previous studies have shown that the 24-h rhythm of locomotor activity in female and male *D. helophoroides* was elevated in the dark phase and reduced in the light phase, and the peak of locomotor activity occurred at 20:30–22:30 during the dark period, while that of adults hardly occurred during light period, indicating that they are nocturnal insects (Lyu et al., 2015b; Lyu et al., 2015c). Further researches showed that increasing the light duration during the dark phase significantly decreased the activity percentage of locomotor, disturbing the normal rhythm of locomotor activity. Moreover, the oviposition ability of females was decreased under light (1, 10, and 100 lx) exposure at night relative to no light (0 lx) at night (Jiang et al., 2023b). In this study, female and male adults exhibited negative phototaxis for LED lights (Figures 5B, D), which is an adaptation strategy to avoid the adverse influence of light exposure.

In the organism response to abiotic stress, a comparative transcriptome analysis was performed using RNA-Seq to examine the cellular responses of plants and animals in response to heavy metals, heat, drought, pathogenesis and insect stress, and up- and downregulated DEGs were identified and analyzed, and potential regulatory mechanisms were speculated or verified under adverse situations (Sun et al., 2022; Wang Z. et al., 2023; Liu et al., 2023). For example, RNA-Seq was used in crayfish experiments under Cu stress, and 4,662 DEGs, including 3,534 upregulated and 1,128 downregulated DEGs, were identified (Wang Z. et al., 2023). In the present study, a total of 1,052 DEGs were identified in the head of *D. helophoroides* following light exposure for 15 and 120 min, and different comparison groups had different up- and downregulation (Figures 5C–I). These results indicated that transcriptional alterations occurred in the head of this beetle during light exposure for 15 and 120 min relative to dark adoption. STEM analysis revealed that 3 clusters (DEGs) significantly changed in male and 4 clusters significantly changed in females (Figures 7D–K), suggesting that differential response strategies may be employed by female and male beetle at different light exposure times. This corroborates the results of behavioral experiments (Figure 2) that showed that trapping rate changes were observed under different light exposures.

Our previous study showed that the locomotor activity percentage of beetles under the light phase was significantly lower than that under the dark phase, suggesting that long-term light exposure can derange temporal adaptation and decrease organism fitness (Jiang et al., 2023b). In the present study, KEGG pathway analysis showed that the lysosome pathway was enriched (Figure 6B), and all of the relative genes in this pathway were downregulated under light exposure for 15 and 120 min relative to dark adaptation, suggesting that the function of this pathway decreased under light exposure. The autophagy lysosome pathway is a major mechanism for degrading intracellular macromolecules, which is also known to protect adult cells against irreversible states (Fujimaki et al., 2019; Lawrence and Zoncu, 2019). In addition, a previous study also showed that reducing lysosomal function drove cells progressively deeper into quiescence depth, and even into a senescence-like irreversibly arrested state (Fujimaki et al., 2019). Reducing lysosomal function may suggest that light illumination may have a negative effect on this nocturnal beetle. Thus, in transcriptome level, the results also showed that female and male adults have a lower or negative phototactic behavioral response rates to light illumination (Figure 4).

In addition, the focal adhesion pathway was also significantly enriched statistically in the 15 and 120 min light exposure groups (female and male) relative to dark adaptation (Figure 6B and 8 C, Supplementary Table S12, *p*_adjust_ < 0.001). Focal adhesions are key modulators of cellular responses to biotic and abiotic stimuli in cell proliferation, differentiation, and motility (Wehrle-Haller, 2012). In crustaceans, focal adhesions have been reported to be involved in Cd, Pb and Cu tolerance through transcriptomic and proteomic approaches (Lu et al., 2019; Jiao et al., 2021; Wang Z. et al., 2023). In crayfish, focal adhesion pathway plays a critical role in the crayfish response to Cu stress and that upregulation of hub genes may increase the survival capacity of crayfish (Wang Z. et al., 2023). However, in our study, genes linked to focal adhesion were significantly downregulated under light exposure for 15 and 120 min relative to dark adaptation (Figure 6B; Supplementary Table S16). In the real environment, there is a significant difference between light stresses and heavy metal stresses. The heavy metal pollutants in aquatic environments are generally widespread. For example, Cu ions are significantly enriched in water, sediment, and cultured fish species around aquaculture farms in the Yangtze River and Taihu Lake, East China (Rajeshkumar et al., 2018; Xiong et al., 2020). Crustacean animals hardly choose the optimal living environment through migration, while nocturnal insects can select a suitable habitat to avoid light exposure through migration. Therefore, crustacean animals may decrease the influence of heavy metals through an upregulated focal adhesion pathway, while the focal adhesion pathway was downregulated in nocturnal insects under light exposure relative to the normal environment (dark adaptation) to impact normal physiological activity.

**FIGURE 10:**
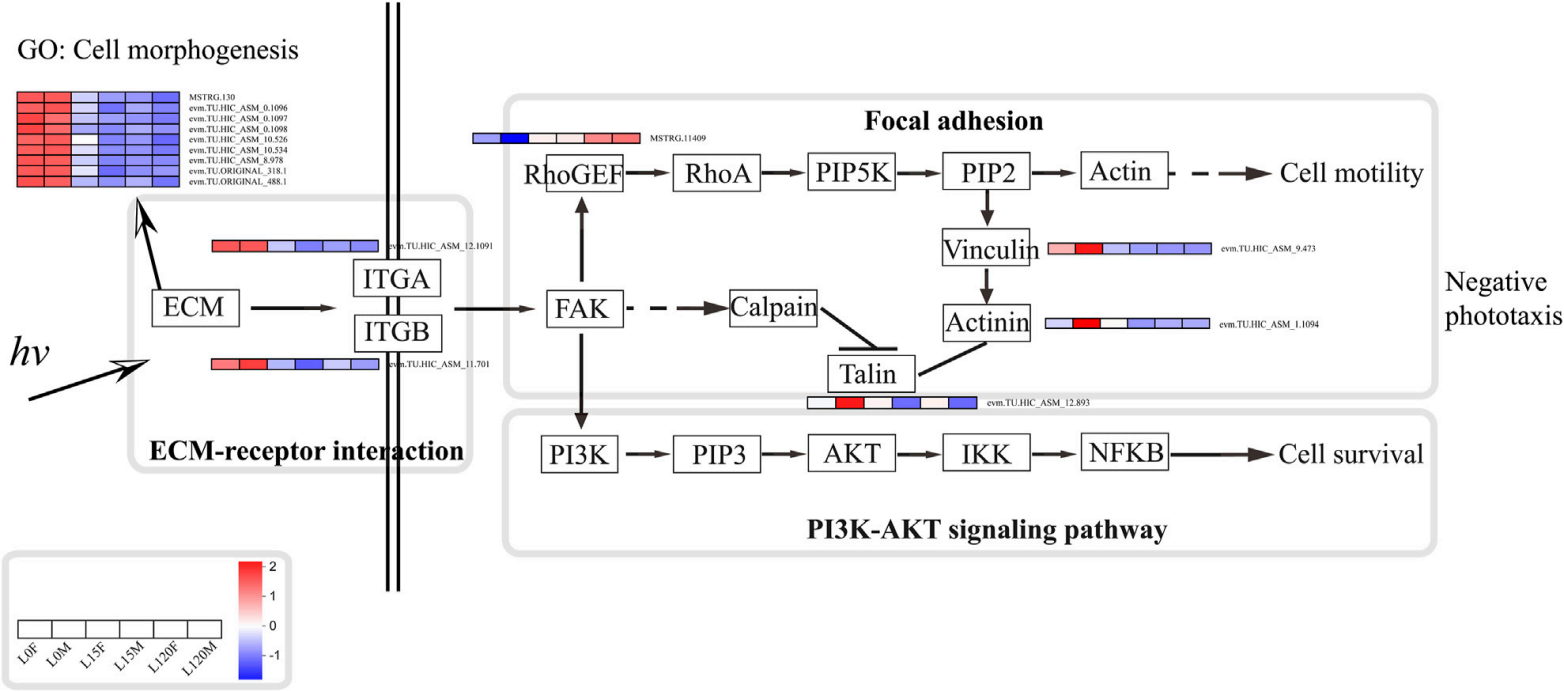
Schematic diagram of the potential response mechanisms of *D. helophorides* involved in light exposure.

Furthermore, focal adhesions are specialized sites within the cell, where mechanical force generated within the cell is transmitted to the surrounding ECM, thus influencing its organization and also contributing to cell migration (Burridge, 2017). The compound eye of insects usually regulates the length and shape of the cone, the cross-sectional areas and shapes of the rhabdoms, and the movement of pigment cells to adapt to changes in light and dark environments (Insausti et al., 2013; Chen et al., 2019). For example, cones of *M. alternatus* were 14.67 ± 0.51 μm in length and formed a conical shape with a narrow and pointed proximal end in the light-adapted state, while cones were much shorter (5.57 ± 0.16 μm) and had a disc-shaped structure with a round and blunt proximal end in the dark-adapted state. The cross-sectional area of the rhabdom decreases by approximately 35% in the light-adapted state relative to that of the dark-adapted state (Wen et al., 2020). Furthermore, photoreceptors have a light/dark adaptation mechanism for adjusting light sensitivity through changes in rhodopsin levels and cellular location in the crepuscular mosquito *Anopheles gambiae* (Moon et al., 2014). In the present study, GO annotation analysis showed that the DEGs were significantly enriched in cell morphogenesis, cell morphogenesis involved in differentiation, basement membrane, and substrate adhesion-dependent cell spreading under light exposure for 15 and 120 min relative to dark conditions (Figure 6A), suggesting some of cell redistribution during change in the light and dark environment. Moreover, DEGs that were enriched in the focal adhesion pathway were mainly distributed in the cell motility and eventually into regulation of actin cytoskeleton (Figure 10; Supplementary Figure S3). For arthropods, rhodopsin was present within the cytoplasmic compartment during daylight timepoints, while it moved to the rhabdomeres only under dark conditions to enhance visual capability in a low light environment (Sacunas et al., 2002; Hu et al., 2012; Moon et al., 2014). Therefore, these DEGs may regulate changes in cone, rhabdom and pigment cell length and shape to enhance light signal recognition ability. Whether these DEGs can regulate changes in cone cells, movement of pigment cells and cross-sectional areas and shapes of the rhabdoms requires further experiments.

Based on the results from this study and other previous studies (Macias-Muñoz et al., 2019), a potential synergistic molecular network can be proposed for the nocturnal beetle *D. helophoroides* in response to light exposure (Figure 10). When light photons were received by photoreceptor cells, genes related to the ECM-receptor interaction pathway are downregulated, such as MSTRG. 130, evm.TU.HIC_ASM_0.1906, 1,097, 1,098, evm.TU.HIC_ASM_10.534, and evm.TU.HIC_ASM_8.978 were downregulated under light exposure (15 and 120 min) relative to dark adaptation (Figure 10). Furthermore, these genes also regulated cell morphogenesis and differentiation to decrease the amount of received light quantum, reducing cellular damage. Subsequently, ITGA (evm.TU.HIC_ASM_12.1091) and ITGB (evm.TU.HIC_ASM_11.701) were downregulated; and then on the one hand, RhoGEF was upregulated to further influence the focal adhesion pathway; on the other hand, PI3K-AKT signaling pathway was regulated, impacting cell motility and survival. Finally, beetles showed negative phototaxis under light exposure.

## Data Availability

RNA sequencing raw data has been deposited in the NCBI Sequence Read Archive (SRA, https://www.ncbi.nlm.nih.gov/sra/, accession numbers SRR25020620 to SRR25020637).
