# Supplementary material for: Integrated behavior and transcriptomic analysis provide valuable insights into the response mechanisms of Dastarcus helophoroides Fairmaire to light exposure
Source: Front Physiol. 2023 Dec 1;14:1250836. doi: 10.3389/fphys.2023.1250836 (PMC10722319; doi:10.3389/fphys.2023.1250836)
Supplement: Supplementary file 1 [file DataSheet1.ZIP › Supplementary material.docx]

**Supplementary Materials:**

**Figures**

Figure S1: Trapping effects of semiochemicals in Cerambycinae and Lamminae. Data are the mean ± SEM from the optimal trapping lure. Trapped ALBs were collected each week, and the other beetles were recorded every 10-14 days. Mp: 4-(n-heptyloxy)butanal and 4-(n-heptyloxy)butanol. pv: linalool, linalool oxide, cis-3-hexen-1-ol, camphene, β-caryophyllene, and 3-carene. hk: cis-3-hexen-1-ol, camphene, linalool, delta-3-carene, and β-caryophyllene. The red line is the reference line.

Figure S2: Temporal expression analysis of the DEGs in the head of *D. helophoroides* following light exposure at 0, 15 min and 120 min using STEM software (male: A, B, C, and D; female: E, F, G, and H).

Figure S3: The detail diagram of pathway from ‘focal adhesion’ pathway. Red frame indicated upregulated genes; green frame indicated downregulated genes.

**Tables**

Table S1: List of primers used in this study.

Table S1: Summary of RNA-sequencing of the heads of *D. helophoroides* in the different light exposure times.

Table S2: The mapping rate of clean reads in each sample for reference genome.

Table S3: The detailed result of KEGG annotation under L120F *vs*. L0F.

Table S4: The detailed result of KEGG annotation under L15F vs. L0F.

Table S5: The detailed result of KEGG annotation under L120M vs. L0M.

Table S6: The detailed result of KEGG annotation under L15M vs. L0M.

Table S7: Summary of the differentially expressed genes (DEGs) in the head of females and males *D. helophoroides* following light exposure.

Table S8: Hub gene analysis in blue modules.

Table S9: Hub gene analysis in turquoise modules.

Table S10: Hub gene analysis in grey modules.

Table S11: the genes from turquoise modules were subjected to KEGG enrichment analysis.

Table S12: the genes from blue modules were subjected to KEGG enrichment analysis.

Table S13. the genes from grey modules were subjected to KEGG enrichment analysis.

Table S14: Downregulated hub gene analysis in four KEGG pathway (‘ECM-receptor interaction’, ‘Focal adhesion’, ‘PI3K-Akt signaling pathway’ and 'Lysosome').

Table S15: The key gene expression differently in the four KEEG pathways (‘ECM-receptor interaction’, ‘Focal adhesion’, ‘PI3K-Akt signaling pathway’ and 'Lysosome').
