# Supplementary material for: Integrated behavior and transcriptomic analysis provide valuable insights into the response mechanisms of Dastarcus helophoroides Fairmaire to light exposure
Source: Front Physiol. 2023 Dec 1;14:1250836. doi: 10.3389/fphys.2023.1250836 (PMC10722319; doi:10.3389/fphys.2023.1250836)
Supplement: Supplementary file 1 [file DataSheet1.ZIP › Figure S1-3.docx]

**Supplementary figures**

**Figure S1.** Trapping effects of semiochemicals in Cerambycinae and Lamminae. Data are the mean ± SEM from the optimal trapping lure. Trapped ALBs were collected each week, and the other beetles were recorded every 10-14 days. Mp: 4-(n-heptyloxy)butanal and 4-(n-heptyloxy)butanol. pv: linalool, linalool oxide, *cis*-3-hexen-1-ol, camphene, *β*-caryophyllene, and 3-carene. hk: *cis*-3-hexen-1-ol, camphene, linalool, delta-3-carene, and *β*-caryophyllene. The red line is the reference line. Data were obtained from the following references: [1-9].





**Figure S2**. Temporal expression analysis of the DEGs in the head of D. helophorides following light exposure at 0, 15 min and 120 min using STEM software (male: A, B, C, and D; female: E, F, G, and H).


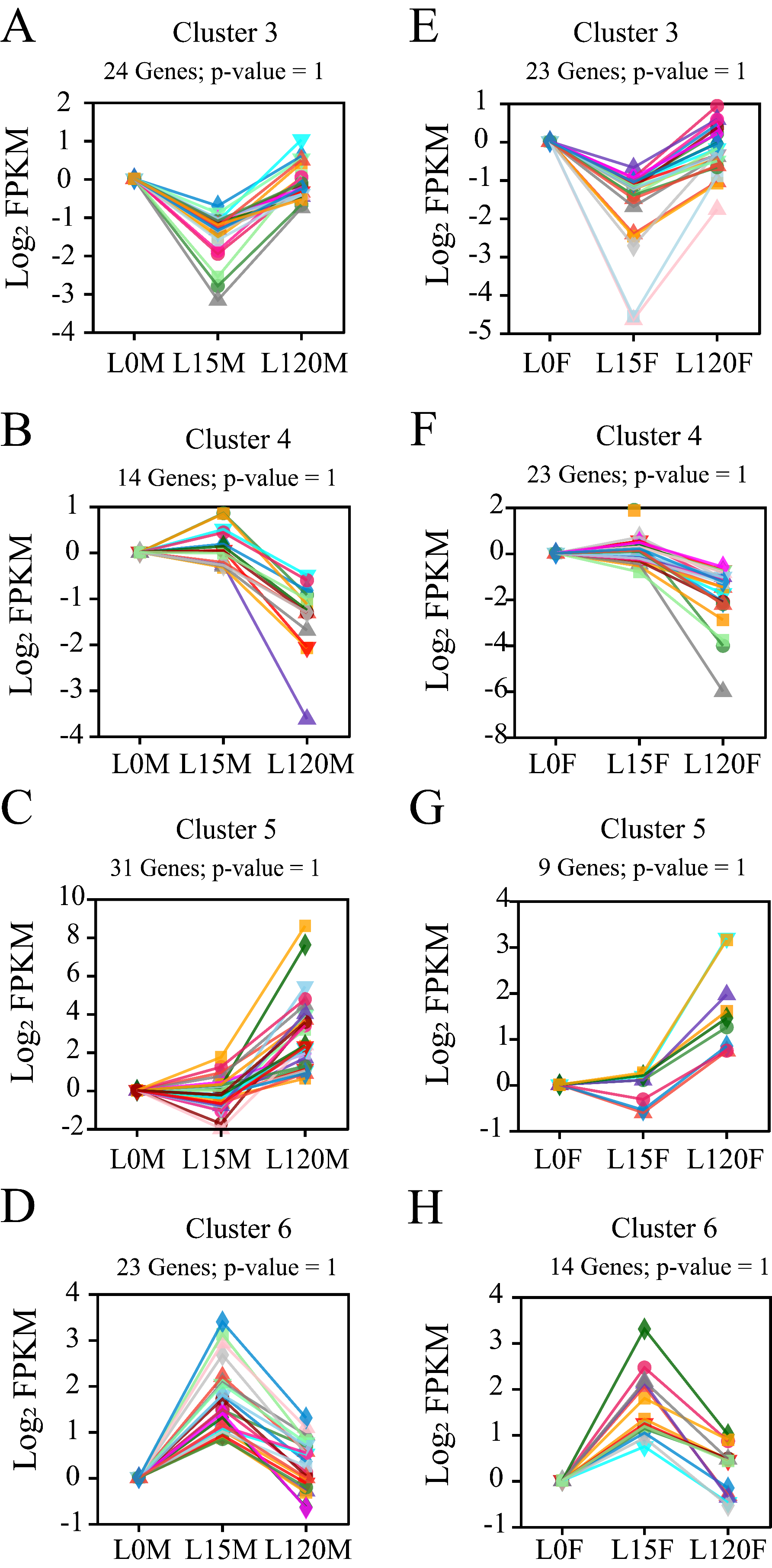


**Figure S3**. The detail diagram of pathway from ‘focal adhesion’ pathway. Red frame indicated upregulated genes; green frame indicated downregulated genes.


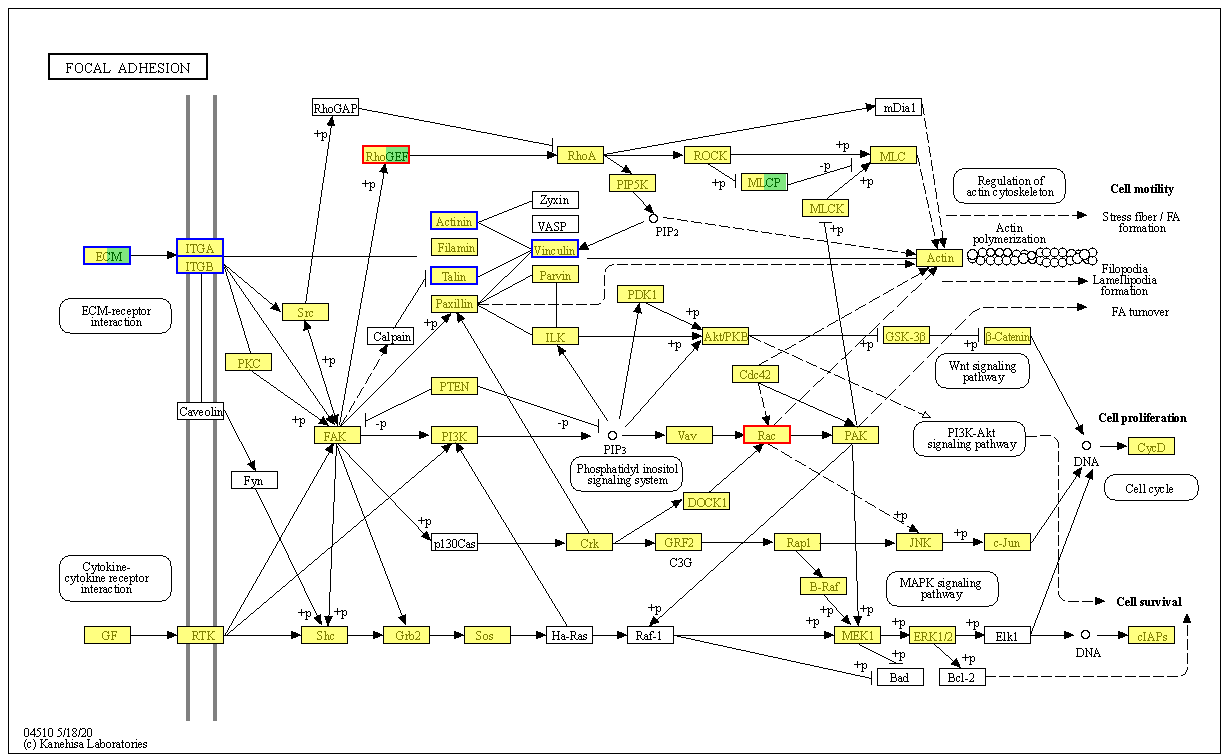


References

1. Nehme, M.E.; Keena, M.A.; Zhang, A.; Baker, T.C.; Xu, Z.; Hoover, K. Evaluating the use of male-produced pheromone components and plant volatiles in two trap designs to monitor Anoplophora glabripennis. *Environ Entomol* **2010**, *39*, 169-176, doi:10.1603/EN09177.

2. Hanks, L.M.; Millar, J.G. Field bioassays of cerambycid pheromones reveal widespread parsimony of pheromone structures, enhancement by host plant volatiles, and antagonism by components from heterospecifics. *Chemoecology* **2013**, *23*, 21-44, doi:10.1007/s00049-012-0116-8.

3. Ray, A.M.; Barbour, J.D.; McElfresh, J.S.; Moreira, J.A.; Swift, I.; Wright, I.M.; Žunič, A.; Mitchell, R.F.; Graham, E.E.; Alten, R.L.; et al. 2,3-Hexanediols as sex attractants and a female-produced sex pheromone for cerambycid beetles in the prionine genus Tragosoma. *J Chem Ecol* **2012**, *38*, 1151-1158, doi:10.1007/s10886-012-0181-z.

4. Wickham, J.D.; Xu, Z.; Teale, S.A. Evidence for a female-produced, long range pheromone of Anoplophora glabripennis (Coleoptera: Cerambycidae). *Insect Science* **2012**, *19*, 355-371, doi:10.1111/j.1744-7917.2012.01504.x.

5. Wickham, J.D.; Harrison, R.D.; Lu, W.; Guo, Z.; Millar, J.G.; Hanks, L.M.; Chen, Y. Generic lures attract cerambycid beetles in a tropical montane rain forest in southern China. *J Econ Entomol* **2014**, *107*, 259-267, doi:10.1603/ec13333.

6. Wickham, J.D.; Millar, J.G.; Hanks, L.M.; Zou, Y.; Wong, J.C.; Harrison, R.D.; Chen, Y. (2R,3S)-2,3-Octanediol, a female-produced sex pheromone of Megopis costipennis (Coleoptera: Cerambycidae: Prioninae). *Environ Entomol* **2016**, *45*, 223-228, doi:10.1093/ee/nvv176.

7. Silva, W.D.; Millar, J.G.; Hanks, L.M.; Bento, J.M. (6E,8Z)-6,8-pentadecadienal, a novel attractant pheromone produced by males of the Cerambycid beetles Chlorida festiva and Chlorida costata. *J Chem Ecol* **2016**, *42*, 1082-1085, doi:10.1007/s10886-016-0742-7.

8. Zhu, N.; Zhang, D.; Wu, L.; Hu, Q.; Fan, J. Attractiveness of aggregation pheromone and host plant volatiles to Anoplophora glabripennis and A. chinensis (Coleoptera: Cerambycidae). *Acta Entomologica Sinica* **2017**, *60*, 421-430, doi:10.16380/j. kcxb. 2017.04.007.

9. Xu, T.; Hansen, L.; Cha, D.H.; Hao, D.; Zhang, L.; Teale, S.A. Identification of a female-produced pheromone in a destructive invasive species: Asian longhorn beetle, Anoplophora glabripennis. *Journal of Pest Science* **2020**, *93*, 1321-1332, doi:10.1007/s10340-020-01229-3.
